# Insights into Heterologous Biosynthesis of Arteannuin B and Artemisinin in *Physcomitrella patens*

**DOI:** 10.3390/molecules24213822

**Published:** 2019-10-23

**Authors:** Nur Kusaira Khairul Ikram, Arman Beyraghdar Kashkooli, Anantha Peramuna, Alexander R. van der Krol, Harro Bouwmeester, Henrik Toft Simonsen

**Affiliations:** 1 Institute of Biological Sciences, Faculty of Science, University of Malaya, Kuala Lumpur 50603, Malaysia; nkusaira@um.edu.my; 2 Centre for Research in Biotechnology for Agriculture (CEBAR), University of Malaya, Kuala Lumpur 50603, Malaysia; 3 Laboratory of Plant Physiology, Wageningen University and Research, Droevendaalsesteeg 1, 6708 PB Wageningen, The Netherlands; arman.beyraghdarkashkooli@wur.nl (A.B.K.); sander.vanderkrol@wur.nl (A.R.v.d.K.); h.j.bouwmeester@uva.nl (H.B.); 4 Bioscience, Wageningen Plant Research, Wageningen University and Research, Droevendaalsesteeg 1, 6708 PB Wageningen, The Netherlands; 5 Department of Biotechnology and Biomedicine, Technical University of Denmark, Søltofts Plads, 2800 Kgs. Lyngby, Denmark; aperamuna@gmail.com; 6 Plant Hormone Biology group, Swammerdam Institute for Life Sciences, University of Amsterdam, 1098 XH Amsterdam, The Netherlands

**Keywords:** artemisinin, *Physcomitrella patens*, sesquiterpenoids, malaria, biotechnology

## Abstract

Metabolic engineering is an integrated bioengineering approach, which has made considerable progress in producing terpenoids in plants and fermentable hosts. Here, the full biosynthetic pathway of artemisinin, originating from *Artemisia annua*, was integrated into the moss *Physcomitrella patens*. Different combinations of the five artemisinin biosynthesis genes were ectopically expressed in *P. patens* to study biosynthesis pathway activity, but also to ensure survival of successful transformants. Transformation of the first pathway gene, *ADS*, into *P. patens* resulted in the accumulation of the expected metabolite, amorpha-4,11-diene, and also accumulation of a second product, arteannuin B. This demonstrates the presence of endogenous promiscuous enzyme activity, possibly cytochrome P450s, in *P. patens*. Introduction of three pathway genes, *ADS-CYP71AV1-ADH1* or *ADS-DBR2-ALDH1* both led to the accumulation of artemisinin, hinting at the presence of one or more endogenous enzymes in *P. patens* that can complement the partial pathways to full pathway activity. Transgenic *P. patens* lines containing the different gene combinations produce artemisinin in varying amounts. The pathway gene expression in the transgenic moss lines correlates well with the chemical profile of pathway products. Moreover, expression of the pathway genes resulted in lipid body formation in all transgenic moss lines, suggesting that these may have a function in sequestration of heterologous metabolites. This work thus provides novel insights into the metabolic response of *P. patens* and its complementation potential for *A. annua* artemisinin pathway genes. Identification of the related endogenous *P. patens* genes could contribute to a further successful metabolic engineering of artemisinin biosynthesis, as well as bioengineering of other high-value terpenoids in *P. patens*.

## 1. Introduction

Artemisinin is a potent malaria drug that is exclusively produced in the plant *Artemisia annua*. The limited production of artemisinin in glandular trichomes of leaves and flowers has led to an extensive cultivation of *Artemisia* plants to meet the needs of the patients. The complex structure of artemisinin makes the chemical synthesis difficult and expensive. Therefore, various efforts have been performed to improve the production of artemisinin in the plant. As alternative, other hosts for heterologous production have been explored, but currently artemisinin production is still mainly based on the use of cultivated plants.

**Scheme 1:**
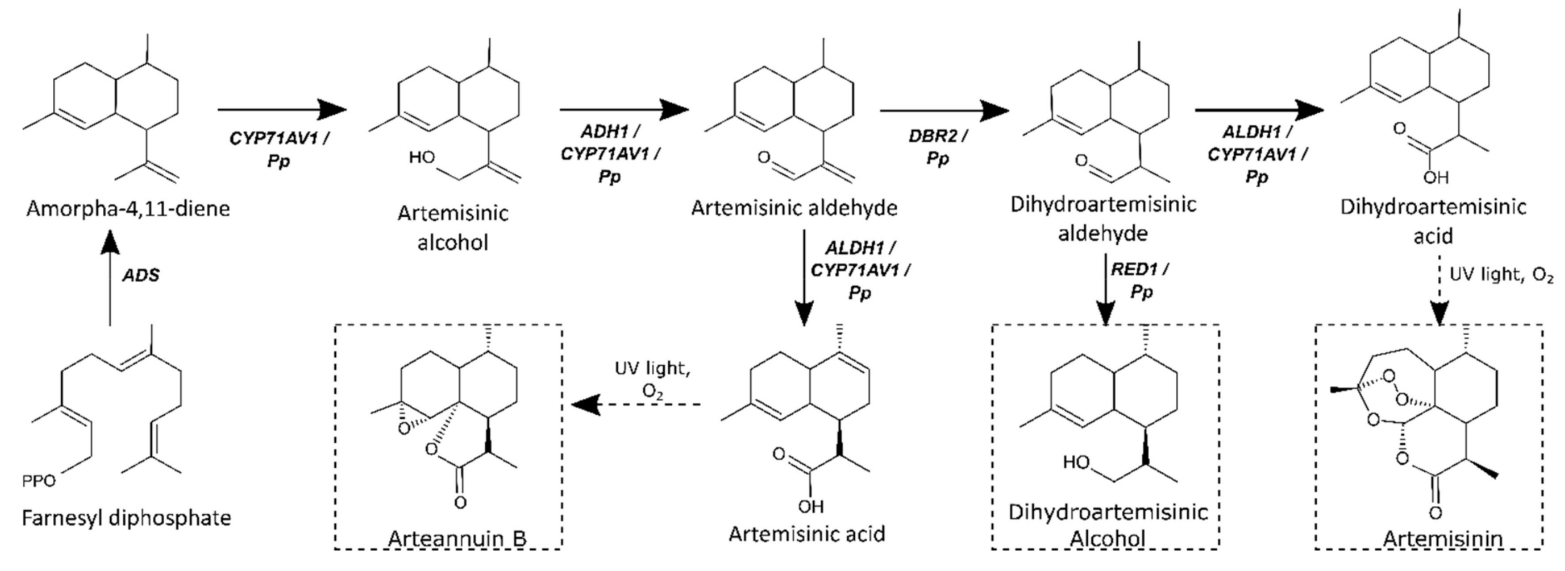
The biosynthetic pathways of artemisinin and arteannuin B in *Artemisia annua*. *Pp* annotation represents possible native *P. patens* enzyme activity. *ADS*, amorphadiene synthase; *CYP71AV1*, amorphadiene oxidase; *ADH1*, alcohol dehydrogenase; *DBR2*, artemisinic aldehyde double-bond reductase; *ALDH1*, aldehyde dehydrogenase 1. The boxes indicate the products of the pathway.

All genes responsible for the biosynthesis of the direct precursor of artemisinin, dihydroartemisinic acid, have been characterized (Scheme 1) [1]. The final conversion of dihydroartemisinic acid to artemisinin is thought to be a light-induced non-enzymatic spontaneous reaction [2]. The first committed biosynthetic step is the cyclization of endogenous farnesyl diphosphate (FPP) to amorpha-4,11-diene by amorpha-4,11-diene synthase (ADS) [3,4,5,6], which is substrate for the next enzyme amorphadiene monooxygenase (CYP71AV1). CYP71AV1 is an important cytochrome P450 enzyme [7] in artemisinin biosynthesis as it catalyses three subsequent oxidations of amorpha-4,11-diene to artemisinic acid, via artemisinic alcohol and artemisinic aldehyde [8]. However, in addition the alcohol dehydrogenase 1 (ADH1, a dehydrogenase/reductase enzyme) has been identified, which specifically produces artemisinic aldehyde from artemisinic alcohol (Scheme 1). This specificity and strong expression in *A. annua* glandular trichomes likely indicates that ADH1 is mainly responsible for biosynthesis of artemisinic aldehyde [9,10]. Artemisinic aldehyde is at a branch point in the bifurcating pathway producing either dihydroartemisinic acid or artemisinic acid [9,10]. In the branch leading to artemisinin, artemisinic aldehyde is reduced to dihydroartemisinic aldehyde by artemisinic aldehyde Δ11(13)-reductase (DBR2) and subsequently is oxidized to dihydroartemisinic acid by an aldehyde dehydrogenase (ALDH1) [11,12,13]. Besides catalysing the oxidation of dihydroartemisinic aldehyde to dihydroartemisinic acid in one branch, in a second pathway branch ALDH1 also catalyses the oxidation of artemisinic aldehyde to artemisinic acid (a reaction also catalysed by CYP71AV1) [7,12]. Another enzyme, dihydroartemisinic aldehyde reductase (RED1) converts dihydroartemisinic aldehyde into dihydroartemisinic alcohol, a “dead end” product, which negatively affects the yield of artemisinin [11]. The final steps in the two branches of the pathway likely involves photo-oxidation of dihydroartemisinic acid and artemisinic acid to artemisinin and arteannuin B, respectively [2,7].

Taking advantage of the elucidated artemisinin pathway, metabolic engineering has been a popular approach to improve the production of artemisinin or its precursors in heterologous hosts such as *E. coli,* yeast and tobacco. A production of amorpha-4,11-diene at 24 g/L was established through the introduction of the MVA pathway and ADS in *E. coli* along with several other modifications [14]. However, expressing plant P450s (such as CYP71AV1) in *E. coli* is not favourable. Therefore *Saccharomyces cerevisiae* (baker’s yeast) was engineered to boost the MVA pathway and ADS through several modifications resulting in a yeast strain producing 153 mg/L amorpha-4,11-diene [8]. Subsequently, CYP71AV1 and a cytochrome P450 reductase (CPR) were introduced resulting in production of up to 100 mg/L of artemisinic acid [8]. The strains were further optimized by adding ADH1, ALDH1 and CYPB5, a native partner of CYP71AV1, that contributed to a significant increase of 25 g/L artemisinic acid via fermentation [9]. Although in these systems complete artemisinin biosynthesis is not accomplished, a 3-step chemical conversion from artemisinic acid to artemisinin has been developed and is currently used in commercial production of artemisinin in combination with yeast fermentation [9,15].

Introducing artemisinin pathway genes in tobacco has also been successful, using both stable and transient expression [16,17,18,19,20,21]. However, in tobacco pathway intermediates are efficiently glycosylated, resulting in low artemisinin yield [16,19]. Attempts have been made to target pathway enzymes to different compartments such as the chloroplast, and Fuentes et al. were able to produce 120 µg/g dry weight (d.w.) artemisinic acid [20], while Malhotra et al. produced 0.8 mg/g d.w. artemisinin in *Nicotiana benthamiana* [22]. All these attempts involved extensive bioengineering of precursor pathways and pathway localization, which is time consuming and with limited success in increasing final yield. Recent work has shown that *Physcomitrella patens* can be a promising heterologous host for artemisinin production, with a high yield of artemisinin after three days, prior to any production enhancements [21,23].

In the present study, various combinations of the pathway genes are assembled to study the biosynthetic route and the interplay with endogenous metabolism as well as ensuring the survival of successful transformants. We observed biosynthetic routes not previously described in the metabolic network of *P. patens* and demonstrate that some endogenous *P. patens* enzymes have promiscuous substrate recognition, which may substitute for some *A. annua* pathway enzyme activities. This provides new insight into *P. patens* metabolism and offers alternative engineering targets for production of artemisinin in this primitive plant and promising heterologous production platform.

## 2. Results and Discussion

### 2.1. Heterologous Expression of Artemisinin Biosynthesis Pathway Genes

The gene encoding the first committed enzyme in the artemisinin biosynthesis pathway (ADS) was introduced into the wild-type (WT) *P. patens.* Integration of the gene was confirmed by PCR on genomic DNA isolated from transformants (Figure S1) and metabolic profiling showed that amorpha-4,11-diene was produced in cultures up to levels up to 200 mg/L [23]. However, localization of the amorpha-4,11-diene remains unclear: is it stored in specific organelles such as lipid bodies or transported out of the cell? Several studies have shown that *P. patens* is able to ectopically produce volatile terpenoids, but the regulation of volatiles production and their potential storage within *P. patens* is yet to be explored [23,24,25]. Besides amorpha-4,11-diene, the transgenic lines expressing *ADS* also accumulated arteannuin B, which is thought to be derived from artemisinic acid through photo-oxidation. This suggest accumulation of artemisinic acid in the transgenic lines and reveals a promiscuous activity of an endogenous oxidative enzyme (or enzymes) such as the cytochrome P450 in *P. patens*, which can catalyse the triple oxidation of amorpha-4,11-diene via artemisinic alcohol and aldehyde to the acid. In *A. annua* these activities are catalysed by CYP71AV1 and ADH1 [8,26]. Although predominant results indicate that most plant P450s are highly specific in their substrate recognition, increasing evidence shows that some plant P450s can be promiscuous in substrate recognition, similar to mammalian P450s [27,28,29,30]. Endogenous *P. patens* oxidative enzymes fully convert amorpha-4,11-diene to artemisinic acid, since the alcohol and aldehyde intermediates were not detected in culture extracts. *P. patens* naturally produces high amounts of *ent*-kaurenoic acid from *ent*-kaurene, which is catalysed by an *ent*-kaurene oxidase (CYP701B1) through three successive oxidations [31,32]. CYP701B1 may therefore be a likely candidate for catalysing the conversion of amorpha-4,11-diene into artemisinic acid in *P. patens.* Other possible candidate enzymes are the numerous cytochrome P450s, ferrodoxin mono-oxygenases, and other oxidoreductases encoded by the *P. patens* genome.

Having established transgenic lines producing a high level of amorpha-4,11-diene, a second transformation introduced the second (*CYP71AV1*) and third (*ADH1*) artemisinin pathway genes. Likewise, the final two artemisinin pathway genes, *DBR2* and *ALDH1* were also introduced into the *ADS* background lines. Having successfully introduced two different sets of 3 artemisinin genes; *ADS-CYP71AV1-ADH1* and *ADS-DBR2-ALDH1* in *P. patens*, the remaining artemisinin pathway genes were introduced into these transgenic lines. This resulted in transgenic lines with all five artemisinin biosynthesis genes in different genomic sequential arrangements. The transgenic lines *ADS-CYP71AV1-ADH1-DBR2-ALDH1* and *ADS-DBR2-ALDH1*-*CYP71AV1-ADH1* were recovered and genotyping showed the presence of all five artemisinin biosynthesis genes in the genome of *P. patens* (Figure S1).

### 2.2. Metabolite Profiling of Transgenic P. patens Lines

In total five different transgenic lines were produced (Table 1). Metabolic profiling of these lines showed that artemisinin was produced in all lines, except for the transgenic line solely expressing *ADS*. The *ADS-CYP71AV1*-*ADH1* line only produced 25% of the artemisinin levels in the *ADS-DBR2-ALDH1* line. This suggests that in the *ADS-DBR2-ALDH1* line the *P. patens* oxidizing enzymes efficiently convert amorpha-4,11-diene to artemisinic aldehyde, which is then converted by DBR2 and ALDH1 to dihydroartemisinic acid (Scheme 1, Table 1). Interestingly, two other metabolites; artemisinic alcohol and dihydroartemisinic alcohol were also detected in the *ADS-DBR2-ALDH1* line. Notably, ADH1 is specific towards artemisinic alcohol [9] and absence of ADH1 in the *ADS-DBR2-ALDH1* lines may explain the accumulation of artemisinic alcohol in this line. The presence of dihydroartemisinic alcohol in the *ADS-DBR2-ALDH1* lines suggests that *P. patens* has an endogenous oxidoreductases similar to *A. annua* RED1 that catalyses the formation of dihydroartemisinic alcohol from dihydroartemisinic aldehyde in *A. annua* [11].

**Figure 1:**
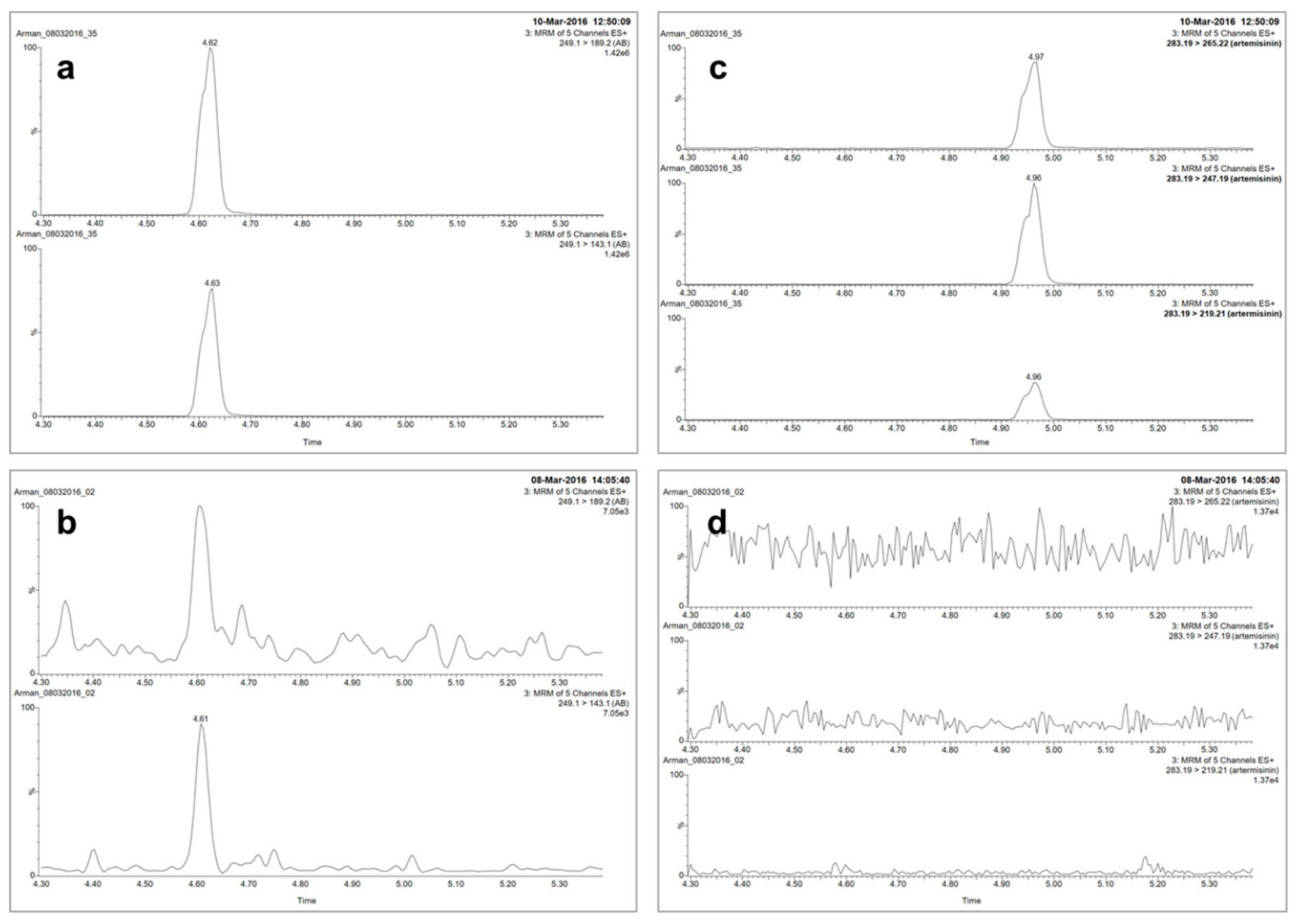
UPLC-MRM-MS analysis of arteannuin B. (**a**) UPLC-MRM-MS of arteannuin B standard fragmented in MRM channels of *m/z* 249.1 > 189.2; 249.1 > 143.1 (**b**) UPLC-MRM-MS analysis of arteannuin B in transformed *P. patens* with *ADS* (RT = 4.61). (**c**) UPLC-MRM-MS of artemisinin standard fragmented in MRM channels of *m*/*z* 283.19 > 219.21; 283.19 > 247.19 and 283.19 > 265.22 (RT = 4.96). (**d**) Demonstration of absence of artemisinin by UPLC-MRM-MS of artemisinin in fragmented in MRM channels of *m/z* 283.19 > 219.21; 283.19 > 247.19 and 283.19 > 265.22 of extracts from transformed *P. patens* with *ADS*. C+D show that artemisinin is not present in the *ADS* only lines of *P. patens*.

Arteannuin B is mostly present in the liquid media (see Figure 1, Table 1) indicating transport capacity for artemisinic acid or arteannuin B to the outside of the cells. Alternatively, this could indicate that accumulation of these compounds is toxic to the cells that then die. The artemisinic aldehyde is at the branch point of the biosynthesis pathway, leading to either dihydroartemisinic acid (precursor of artemisinin) or artemisinic acid (precursor of arteannuin B) (Scheme 1). While *DBR2* catalyses the conversion of artemisinic aldehyde toward artemisinin production, *ALDH1* and *CYP71AV1* or *P. patens* hydroxylases catalyse the formation of artemisinic acid.

Our transgenic *P. patens* lines were grown under constant (24 h) high light intensity. Thus, all the produced artemisinic acid or dihydroartemisinic acid are presumably photo-chemically converted into arteannuin B and artemisinin, respectively and neither of the two acids were detected in our study.

The accumulation of arteannuin B was correlated with the amount of artemisinin. Transformants with higher levels of arteannuin B, also accumulates higher levels of artemisinin. For instance, *ADS-DBR2-ALDH1* accumulates most artemisinin as well as arteannuin B.

The accumulation of artemisinin in the *ADS-CYP71AV1*-*ADH1* line shows that *P. patens* has enzymes with similar activities as DBR2 and ALDH1 from *A. annua*. The lower artemisinin accumulation in the *ADS-CYP71AV1*-*ADH1* line suggests that the affinity of the endogenous *P. patens* enzyme for the pathway intermediates may not be as good as for *A. annua* DBR2 and ALDH1.

Although presence of endogenous enzyme activity might contribute to the accumulation of artemisinin, only arteannuin B was detected in the *ADS* expressing line (see Figure 1). One reason could be that the *P. patens* hydroxylases and oxidoreductases has lower affinity towards the heterologous substrates than the pathway enzymes, CYP71AV1, DBR2 and ALDH1. For example, higher levels of artemisinin was detected when CYP71AV1 was expressed (*ADS-CYP71AV1-ADH1*), which should accumulate artemisinic aldehyde, but this is catalysed into dihydroartemisinic acid by native hydroxylases and oxidoreductases. 

Dihydroartemisinic acid spontaneously transform into artemisinin when exposed to light. Meanwhile, in the *ADS*-only line amorpha-4,11-diene accumulates and here the native hydroxylases might favour reactions resulting in a final accumulation of arteannuin B via artemisinic acid (see Scheme 1 for the pathway).

Unlike in other heterologous plants e.g., *Nicotiana benthamiana* [16], no glycosylated and/or glutathione conjugates of the artemisinin biosynthesis intermediate related products were detected in the transgenic *P. patens* lines [23]. The absence of glycosylated products could be due to the much lower number of genes encoding putative glycosyltransferases in *P. patens,* compared to that in higher (vascular) plants [33]. This could be an important feature of *P. patens* to favour full pathway activity toward the accumulation of the two products artemisinin and/or arteannuin B.

### 2.3. Analysis of Artemisinin Pathway Gene Expression Profiles

**Figure 2:**
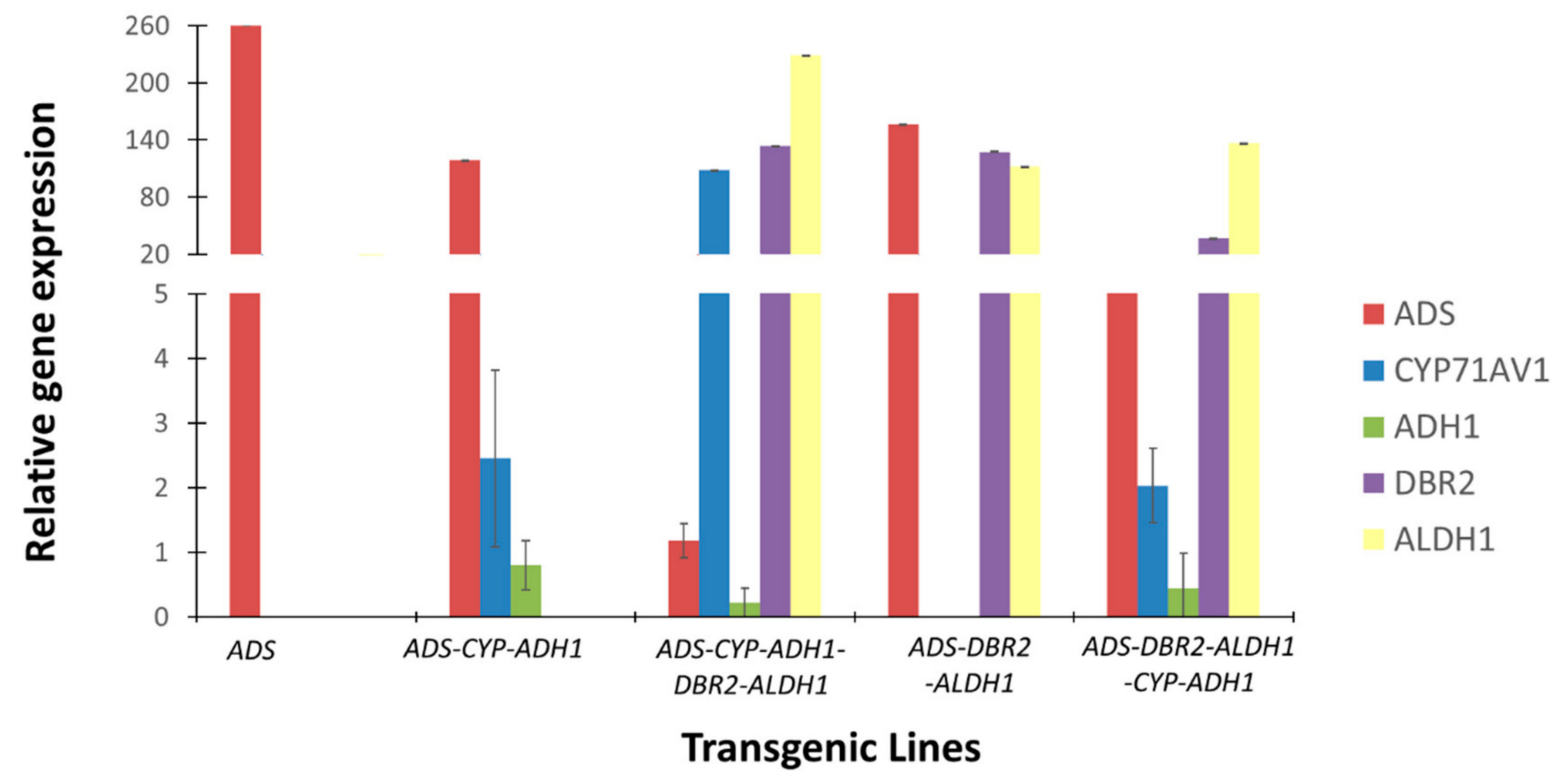
Relative expression of artemisinin pathway genes (*ADS, CYP71AV1, ADH1, DBR2, ALDH1*) in the five transgenic *P. patens* lines. Error bars are shown as SE (n = 3).

Analysis of the artemisinin biosynthetic gene expression profile was performed to investigate the correlation between gene expression and metabolite production in the transgenic lines (Figure 2). The expression of the first committed enzyme, ADS was the highest when it was introduced alone, and decreased with the increasing number of genes introduced; *ADS* > *ADS-CYP71AV1-ADH1* > *ADS-CYP71AV1-ADH1-DBR2-ALDH1*. A similar pattern was observed in the expression of the other constructs with *ADS* > *ADS-DBR2-ALDH1* > *ADS-DBR2-ALDH1-CYP71AV1-ADH1*. For *CYP71AV1*, there was no significant difference in expression between the *ADS-CYP71AV1-ADH1* and *ADS-DBR2-ALDH1-CYP71AV1-ADH1* lines. However, the expression level was 100 fold higher in the *ADS-CYP71AV1-ADH1-DBR2-ALDH1* line showing that higher amount of the enzyme could be present for the higher production of artemisinin. *ADH1* on the other hand exhibited a low expression pattern in all transgenic lines, suggesting its limited contribution to the overall artemisinin pathway in *P. patens*.

Overall, *ADS-DBR2-ALDH1* shows higher gene expressions for all three genes compared to the other transgenic lines and this correlates positively with the product levels. The expression level of the *ADS* gene in *ADS-DBR2-ALDH1* is the second highest, after the *ADS* only expressing line, which may lead to abundant amorpha-4,11-diene to be catalysed by *DBR2* into artemisinic aldehyde and subsequently into dihydroartemisinic acid by *ALDH1*. However, in addition, endogenous *P. patens* hydroxylases and *ALDH1* efficiently catalyse the formation of artemisinic acid, hence contributing to higher accumulation of arteannuin B than artemisinin (Table 1). The transgene expression levels in all the lines expressing *DBR2* and *ALDH1* correlate well with their end product profiles (Figure 2, Table 1). Similarly, lines *ADS-CYP71AV1*-*ADH1* and *ADS-DBR2-ALDH1-CYP71AV1*-*ADH1*, which show lower expression of *CYP71AV1,* also show lower levels of artemisinin. Results thus suggest that not only higher affinity for substrates but also abundance of the active *A. annua* enzymes has a positive impact on artemisinin levels. Improving expression and protein levels even further may therefore be targets for future research.

The expression of *DBR2* and *ALDH1* was relatively high and correlates with the amount of metabolite produced. Studies on the artemisinin pathway gene expression in different *A. annua* chemotypes: the high artemisinin producer (HAP) and low artemisinin producer (LAP) as well as the *Nicotiana benthamiana* transiently expressing the artemisinin biosynthetic pathway genes show that the expression of DBR2 is significantly higher in the HAP varieties which is similar to the gene expression pattern found in *P. patens* [16,34]. It is evident that *DBR2* and *ALDH1* appear to be of a great importance in elevating artemisinin production in *P. patens, A. annua* and other heterologous plant-based systems [16,20,34,35].

### 2.4. Lipid Body Formation in Transgenic P. patens

**Figure 3:**
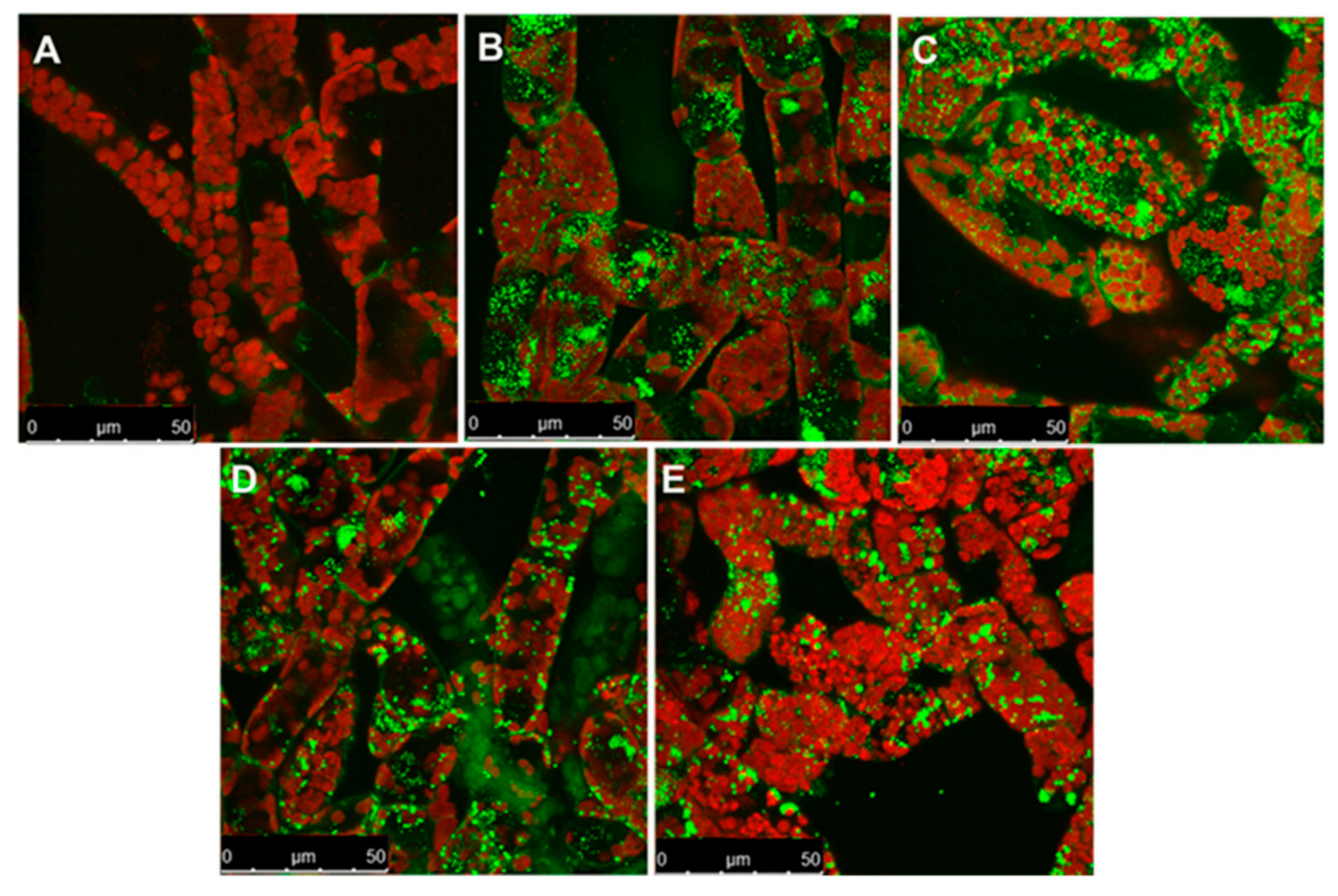
Projections of 8 day old moss obtained by confocal microscopy showing the accumulation of lipid bodies (green spots, stained with BODIPY) in wild type (**A**) and transgenic moss bearing (**B**) *ADS* (**C***) ADS-CYP71AV1-ADH1* (**D**) *ADS-DBR2-ALDH1* (**E**) *ADS-CYP71AV1-ADH1-DBR2-ALDH1*. Red color represent chlorophyll autofluorescence.

*P. patens* utilizes lipid bodies (LBs) in its life cycle [36] and because of the hydrophobic nature of artemisinin, we investigated whether ectopic expression of the artemisinin pathway in transgenic *P. patens* favours LB formation by LB staining with BODIPY. Confocal microscopy observations confirmed that abundant and large LBs are present in all transgenic lines (Figure 3). Formation of these LBs in response to production of potentially toxic compounds could indicate an alternative phytotoxic defence mechanism in *P. patens* prior to the development of alternative detoxification strategies through glycosylation as in higher plants. Glycosylation and modification by glutathione of pathway intermediates are the biggest competitor for production of artemisinin and other sesquiterpenes in heterologous plant expression systems. The absence of such detoxification mechanisms and induction of potential sequester structures like LBs in *P. patens* make this organism a potential valuable novel tool for production of artemisinin or other valuable terpenes. To address this, further research on the mechanism of lipid body formation and identification of LB composition in the transgenic *P. patens* will be valuable for an overall understanding on *P. patens* metabolic responses to heterologously produced metabolites.

## 3. Materials and Methods

### 3.1. Plant Material and Growth Conditions

*P. patens* (Gransden ecotype, International Moss Stock Center #40001) was grown on solid and liquid PhyB media under sterile conditions, at 25 °C with continuous 20–50 W/m^2^ light intensity [37].

### 3.2. DNA Fragments and Genes

The Pp108 locus homologous recombination flanking regions were amplified from genomic DNA of *P. patens*. The *ADS* gene was a kind gift from Assoc. Prof. Dae Kyun Ro, University of Calgary, Calgary, AB, Canada. The synthetic genes of *CYP71AV1* (DQ268763), *ADH1* (JF910157.1), *DBR2* (EU704257.1), and *ALDH1* (FJ809784.1) were synthesized by GenScript (city, state abbrev USA) according to the *P. paten* codon usage. The synthetic genes was linked with a peptide linker LP4/2A from *Impatiens balsamina* and foot-and-mouth-disease virus (FMDV); *CYP71AV1-*LP4/2A*-ADH1*, *DBR2-*LP4/2A*-ALDH1*. The Ubiquitin promoter and Ubiquitin terminator from *Arabidopsis thaliana* (CP002686.1) synthetic genes were also synthesized by GenScript. The Maize Ubiquitin 1 promoter, *OCS* terminator and G418 selection cassettes was obtained from the pMP1355 vector, a kind gift from Professor Mark Estelle, University of California San Diego, San Diego, CA, USA.

### 3.3. Transformation Procedures

A detailed description of moss transformation has been previously published [37,38]. Five to seven day old *P. patens* cultures (from last blending) was harvested and digested with 0.5% DriselaseR enzyme solution in 8.5% mannitol (D9515, Sigma Aldrich) followed by incubation at room temperature for 30 to 60 min. The digested sample was then filtered through a 100 μm pored mesh-filter and the protoplast was collected by centrifugation at 150–200× *g* for 4 min with slow breaking. The pellet was washed twice with the protoplast wash solution (8.5% mannitol, 10 mM CaCl_2_) and the protoplast density was measured using a hemocytometer before suspending in MMM solution (9.1% D-mannitol, 10% MES and 15mM MgCl_2_) to a concentration of 1.6 × 106 protoplasts/mL. 300 μL of the protoplast suspension and 300 μL of PEG solution were added to a 15 mL tube containing 10 μg total DNA and incubated at 45 °C for 5 min and another 5 min at room temperature. 8.5% D-mannitol (300 μL) was then added five times and dilutions with 1 mL of 8.5% D-mannitol another five times. The transformed protoplasts were collected by centrifugation, resuspended in 500 μL of 8.5% d-mannitol and 2.5 mL of protoplast regeneration media (top layer; PRMT). One ml of the mixture was distributed on three plates containing protoplast regeneration media (bottom layer; PRMB) overlaid with cellophane. The plates were incubated in continuous light for 5 to 7 days at 25 °C. The cellophane and regenerating protoplasts was then transferred to PhyB media containing the appropriate selection marker for two weeks, before transferring on PhyB media without antibiotics for another 2 weeks and later transferred back to the final antibiotic selection to confirmed stable transformants.

The first committed precursor of artemisinin biosynthesis pathway, *ADS* was introduced into wildtype *P. patens* at the designated neutral locus Pp108. The transformed lines were selected on regeneration medium with geneticin (G418) for two rounds of selection. Next, we transformed the second and third: *CYP71AV1*-LP4/2A-*ADH1* as well as the fourth and fifth; *DBR2*-LP4/2A-*ALDH1* genes respectively into the *ADS*-expressing transgenic line. Both transformations are targeted to replace the previously transformed G418 selection marker with the new selection of hygromycin. Having successfully introduced three artemisinin genes; *ADS-CYP71AV1-ADH1* and *ADS-DBR2-ALDH1* in *P. patens*, we next completed the pathway with addition of the remaining artemisinin genes into both transgenic lines. For this transformation, the previously removed G418 selection marker was used again and hygromycin was targeted for recombination such that this selection marker was removed.

### 3.4. PCR, DNA Purification and Concentration

All DNA fragments were amplified with PhusionR High-Fidelity DNA Polymerase (New England Biolabs, County Road Ipswich, MA, USA). PCR conditions and annealing temperatures were modified depending on primers and templates used in the reaction. PCR reactions using plasmid DNA as template were digested with DpnI (NEB, County Road Ipswich, MA, USA) for 1 h at 37 °C followed by inactivation at 65 °C for 20 min to lower background after transformation. PCR products were purified using QIAquick PCR Purification Kit (Qiagen GmbH, Strasse 1, Hilden, Germany). The DNA fragments for transformations were concentrated via ethanol precipitation to a final concentration of ~1 μg/μL, determined using NanoDrop2000 (Thermo Fisher Scientific, Waltham, MA, USA). The primers used are listed in Table S1.

### 3.5. Metabolite Profiling

#### 3.5.1. UPLC-MRM-MS Analysis

Fresh moss samples were harvested, snap-frozen and ground into a fine powder. Samples of 3000 mg were extracted with 3 mL citrate phosphate buffer, pH 5.4, followed by vortexing and sonication for 15 min. One mL of Viscozyme (V2010, Sigma) was added and samples were incubated at 37 °C. The whole mixture was then extracted three times with 3 mL ethyl acetate and concentrated to a volume of 1 mL and stored at −20 °C. For liquid culture extracts, 500 mL of liquid culture was harvested, passed through a filter paper and extracted with 200 mL of ethyl acetate in a separation funnel. Ethyl acetate was concentrated to a volume of 1 mL and stored at −20 °C. Ethyl acetate of both liquid culture and moss sample extracts were then dried under a flow of N_2_ and resuspended into 300 µL of 75% MeOH:H_2_O (*v*:*v*). Extracts were passed through a 0.45 µm membrane filter (Minisart^®^ RC4, Sartorius, Germany) before analysis. Artemisinin and artemisinin biosynthesis pathway intermediates were measured in a targeted approach by using a Waters Xevo tandem quadrupole mass spectrometer equipped with an electrospray ionization source and coupled to an Acuity UPLC system (Waters), essentially as described [16]. For A BEH C18 column (100 × 2.1 mm × 1.7 µm; Waters) was used for chromatographic separation by applying a water:acetonitrile gradient. The gradient started from 5% (*v*/*v*) acetonitrile in water with formic acid [1:1000 (*v*/*v*)] for 1.25 min, was raised to 50% in 2.35 min and was raised to 90% at 3.65 min. This was kept for 0.75 min before returning to the 5% acetonitrile/water (*v*/*v*) with formic acid [1:1000 (*v*/*v*)] by using a 0.15 min gradient. The same solvent composition was used to equilibrate the column for 1.85 min. The flow rate was 0.5 mL/min and the column temperature was maintained at 50 °C. Injection volume was set to 10 µL. Desolvation and cone gas flow were set to 1000 and 50 L/h and the mass spectrometer was operated in positive ionization mode. Capillary voltage was set at 3.0 kV. Desolvation and source temperatures were set at 650 and 150 °C, respectively. The cone voltage was optimized for all metabolites using the Waters IntelliStart MS Console. Fragmentation by collision-induced dissociation was done in the ScanWave collision cell using argon. Multiple Reaction Monitoring (MRM) was used for detection and quantification of artemisinin and the other compounds. MRM transitions for artemisinin and pathway intermediates measurement settings were optimized for MRM channels, which are presented in Table S2. Targeted analysis of the fragmentation pattern of authenticated standard was optimized for each of the target compounds. For artemisinin three parent(/daughter) ions (expressed as channels) was obtained and for arteannuin B two channels were identified, as previously described [16,23]. The presence or absence of each compound in samples were checked by comparing the RT of compounds in standard mix with samples. As an additional quality measure the ratio between peak intensity of each compound’s channels (e.g., for artemisinin the ratio of 283.19 > 219.21 to 283.19 > 247.19 in samples should be the same as the ratio in what has been measured in artemisinin standard) were checked which was the same in both standards and the identified compounds in samples. Retention time of each compound (positive ionization mode) is presented in Table S3. Artemisinin and dihydroartemisinic acid were gift from Dafra Pharma (Belgium). Other precursors were synthesized from dihydroartemisinic acid by Chiralix (Nijmegen, the Netherlands) which was checked by NMR (>98% purity). External calibration curves were measured by using reference standards.

#### 3.5.2. LC-QTOF-MS for Analysis of Conjugated Artemisinin Pathway Intermediates

For the artemisinin pathway intermediates glycosides and conjugations, 100 mg of fresh *P. patens* tissue was ground in liquid nitrogen and extracted with 300 µl MeOH:formic acid [1000:1 (*v*/*v*)]. Samples were briefly vortexed and sonicated for 15 min, followed by 15 min centrifugation at 13,000× *g*. Extracts were passed through a 0.45 µm membrane filter (Minisart^®^ RC4, Sartorius) before analysis on a Water alliance 2795 HPLC connected to a QTOF Ultima V 4.00.00 mass spectrometer (Waters MS Technologies). The mass spectrometer was operated in negative ionization mode. A precolumn of 2.0 × 4 mm (Phenomenex, Denmark) was connected to the C18 analytical column (Luna 3 µm C18/2 100A; 2.0 × 150 mm; Phenomenex). Degassed eluent A and B were HPLC-grade water:formic acid [1000:1 (*v*/*v*)] and acetonitrile:formic acid [1000:1 (*v*/*v*)], respectively. The flow rate was 0.19 mL/min. The HPLC gradient started from 5% eluent B and linearly increased to 75% in 45 min. After that, the column was equilibrated for 15 min with 5% eluent B. 5 µL of each sample was used for injection.

### 3.6. Lipid Bodies Staining and Microscopy

Equal amount of 14 days old liquid grown cells were suspended in PBS pH 7.4 buffer and stained with a final concentration of 0.5 µg/mL of BIODIPY 505/515 (Invitrogen Molecular Probes, Thermo Fisher). Cells were incubated in the dark for 15 min and visualized with a Leica LAS AF confocal laser microscope. Lipid bodies were visualized with a 488 nm laser excitation line and a 510–530 nm emission window. Chloroplasts were visualized using the same laser line and a 650–700 nm excitation window. Z stacks were performed on each image with a line average of 4 and combined using maximum projection into a single image, and image was visualized with ImageJ [39].

### 3.7. Expression Profiling in P. patens

100 mg of one week old moss tissue was extracted by RNeasy Plant Mini Kit (Qiagen 74904) according to the protocol provided. The samples were treated with DNase I (Sigma AMPD1) to remove remaining genomic DNA. The RNA quality and concentration was determined by Nanodrop2000 Spectrophotometer (Thermo Fisher Scientific). cDNA synthesis was performed with 500 ng RNA samples using SuperScript III First-Strand Synthesis System for RT-PCR kit (18080-051, Life Technologies, Denmark). Real time quantitative PCR was performed using QuantiFast ^®^ SYBR ^®^ Green PCR (Qiagen, Denmark) according to the protocol provided and run at 95 °C for 5 min, 40 cycles at 95 °C for 10 s followed by 60 °C for 30 s on a CFX Connect Real Time PCR Detection System (BioRad, Denmark). The qPCR was performed with three biological replicates for each sample and three technical replicates for each biological sample. Primers used are listed in (Table S1). Efficiencies of all primers were estimated by generating a standard curve via cDNA serial dilutions using this formula E = 10^−1/slope^ − 1. E values of the primer pairs ranged between 93 to 101% (efficiency between 90 and 110% are acceptable). *P. patens* β-actin was used as the reference gene and the transcripts level was calculated as follows: ∆CT = CT(GOI) − CT(Actin), ∆∆CT was normalized using ∆CT and the relative change in gene expression is calculated by 2^−∆∆CT^ method [40].

## 4. Conclusions

Here we show that the anti-malaria drug, artemisinin, can be produced in *P. patens* with either complete or partial introduction and expression of the artemisinin pathway genes. The results demonstrate that *P. patens* expresses endogenous enzymes with similar activity to that of the artemisinin biosynthesis pathway in *A. annua.* This possibly affects the accumulation of artemisinin and arteannuin B. Knocking out the endogenous oxidizing enzyme(s) responsible for the conversion of amorpha-4,11-diene into artemisinic acid could possibly positively affect the yield of artemisinin as it could stimulate the flux towards dihydroartemisinic aldehyde. This work provides novel insights into the metabolic machinery of *P. patens* and shows it has enzymes with activities similar to those that catalyse the artemisinin pathway in *A. annua*. Discovery of these enzymes and the encoding genes may contribute not only to successful metabolic engineering of artemisinin biosynthesis in *P. patens*, but also to the engineering of other high-value terpenes in *P. patens*. Enzymes with promiscuous activity can be of high value for any synthetic biology adventure since they can be used for many purposes. They also shed light on general enzyme activity for the specific classes of enzymes. Work is ongoing to discover these enzymes.

## Figures and Tables

**Table 1:** Quantification of artemisinin, artemisinin intermediates and arteannuin B produced in transgenic *Physcomitrella patens* and the moss culture liquid media (from 3 weeks moss culture, average of two cultures). The content in the liquid media represents the amount of molecules that have been excreted from the moss cells.

	ADS	ADS-CYP71AV1-ADH1	ADS-CYP71AV1-ADH1-DBR2-ALDH1	ADS-DBR2-ALDH1	ADS-DBR2-ALDH1-CYP71AV1-ADH1
Content in culture liquid media (without moss)	(µg/g FW)	(µg/g FW)	(µg/g FW)	(µg/g FW)	(µg/g FW)
Artemisinic alcohol	^ND^	^ND^	^ND^	^ND^	^ND^
Dihydroartemisinic alcohol	^ND^	^ND^	^ND^	0.09	^ND^
Arteannuin B	1.05	0.04	0.09	1.74	^ND^
**Content in dried moss tissue**	**(mg/g DW)**	**(mg/g DW)**	**(mg/g DW)**	**(mg/g DW)**	**(mg/g DW)**
Artemisinin	^ND^	0.01	0.03	0.04	0.01
Artemisinic alcohol	^ND^	^ND^	^ND^	0.13	^ND^
Dihydroartemisinic alcohol	^ND^	^ND^	^ND^	0.07	^ND^

ND, not detected.

## Supplementary Materials

The following are available online. Figure S1 shows the Genotyping of the moss, Figures S2 and S3 show UPLC-MRM-MS analysis of artemisinin and arteannuin B, respectively from the different P. patens transgenic lines. Table S1 shows primers used in the study and Table S2 is the optimized MRM transition settings for UPLC-MRM-MS.
