# Supplementary material for: Insights into Heterologous Biosynthesis of Arteannuin B and Artemisinin in Physcomitrella patens
Source: Molecules. 2019 Oct 23;24(21):3822. doi: 10.3390/molecules24213822 (PMC6864739; doi:10.3390/molecules24213822)
Supplement: Supplementary file 1 [file molecules-24-03822-s001.pdf]

Supplementary data for:

Insights into heterologous biosynthesis of arteannuin B and artemisinin in *Physcomitrella patens*

Nur Kusaira Khairul Ikram[1,4], Arman Beyraghdar Kashkooli[3,5], Anantha Peramuna[2], Alexander R. van der Krol[3], Harro Bouwmeester[3,6] and Henrik Toft Simonsen[2]*

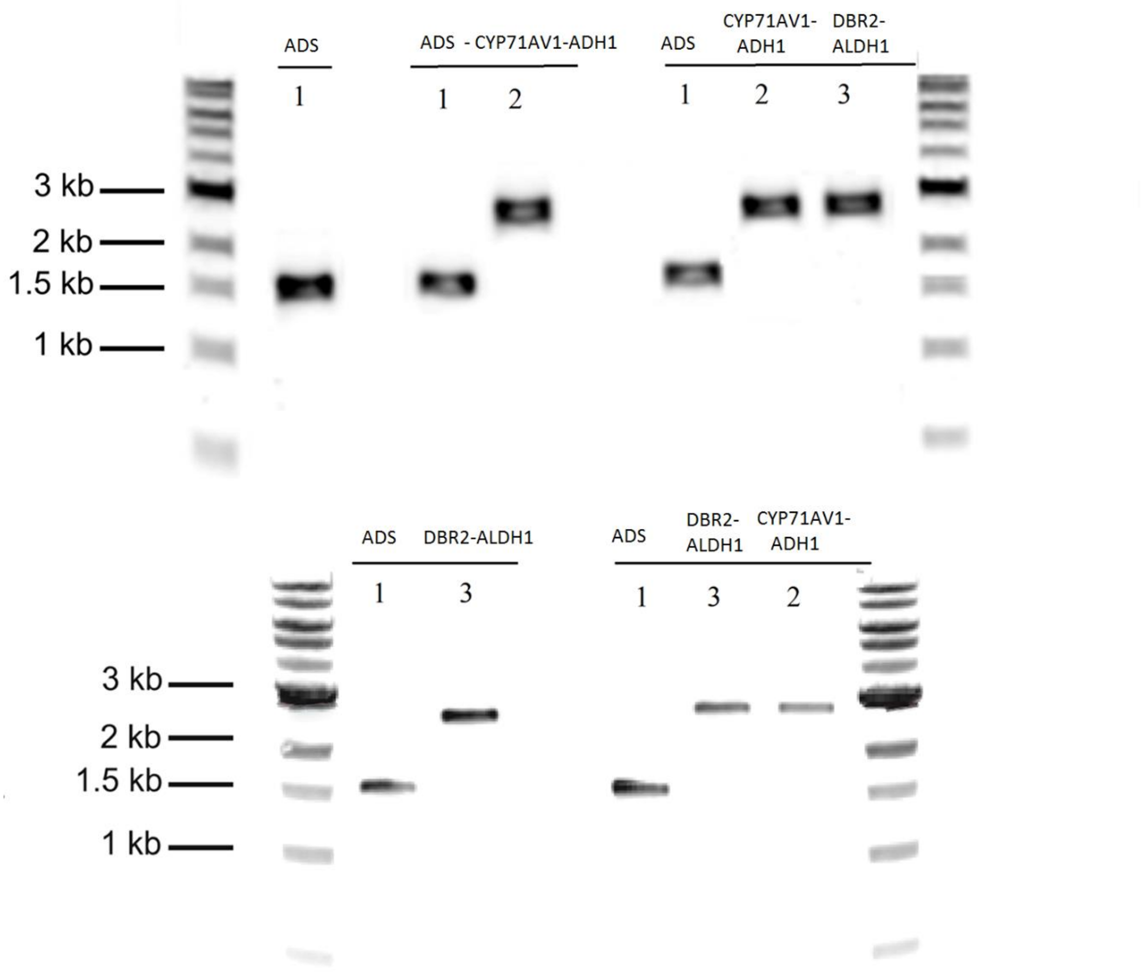

Supplemental Figure S1. Genotyping of the moss lines via PCR using gene-specific primers of 1. *ADS*, 1641bp 2. *CYP71AV1-ADH1*, 2709bp and 3. *DBR2-ALDH1* (2748bp). Each transgenic line is shown for constructs *ADS*; *ADS-CYP71AV1-ADH1*; *ADS-CYP71AV1-ADH1-DBR2-ALDH1*; *ADS-DBR2-ALDH1* and *ADS DBR21-ALDH-CYP71AV1-ADH1*. The PCR products were sequence-verified.

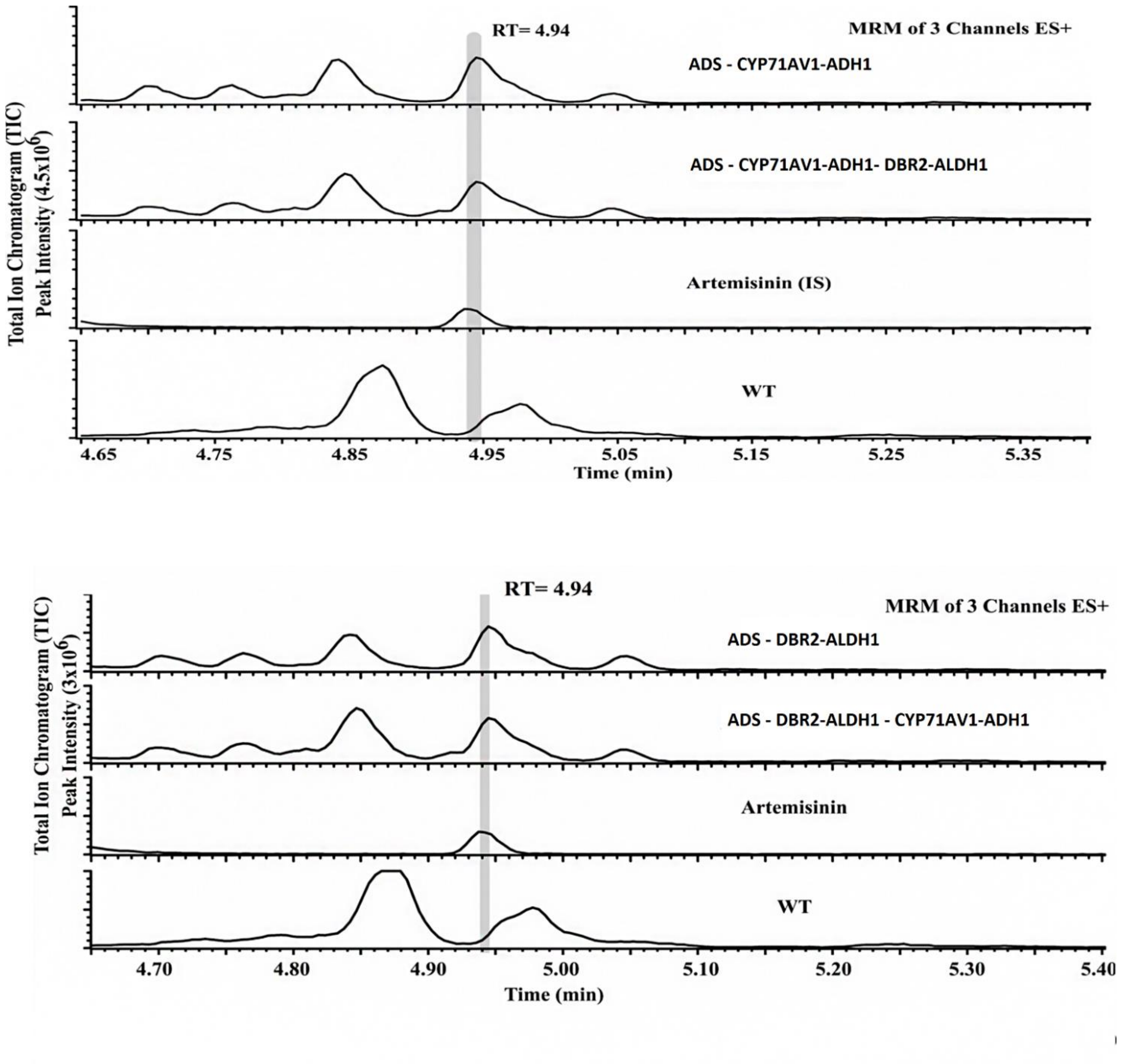

Supplemental Figure S2. UPLC-MRM-MS analysis of artemisinin from various constructs introduced in *P. patens* (*ADS-CYP71AV1-ADH1*; *ADS-DBR21-ALDH*; *ADS-CYP71AV1-ADH1-DBR2-ALDH1*; *ADS-DBR21-ALDH-CYP71AV1-ADH1*), an internal standard (IS) and WT as control with retention time (RT). TIC represents the sum of MRM channels used for the detection of artemisinin: *m/z* 283.19>219.21; 283.19>247.19 and 283.19>265.22. For the full identification of artemisinin production *P. patens* please see previously published results [23].

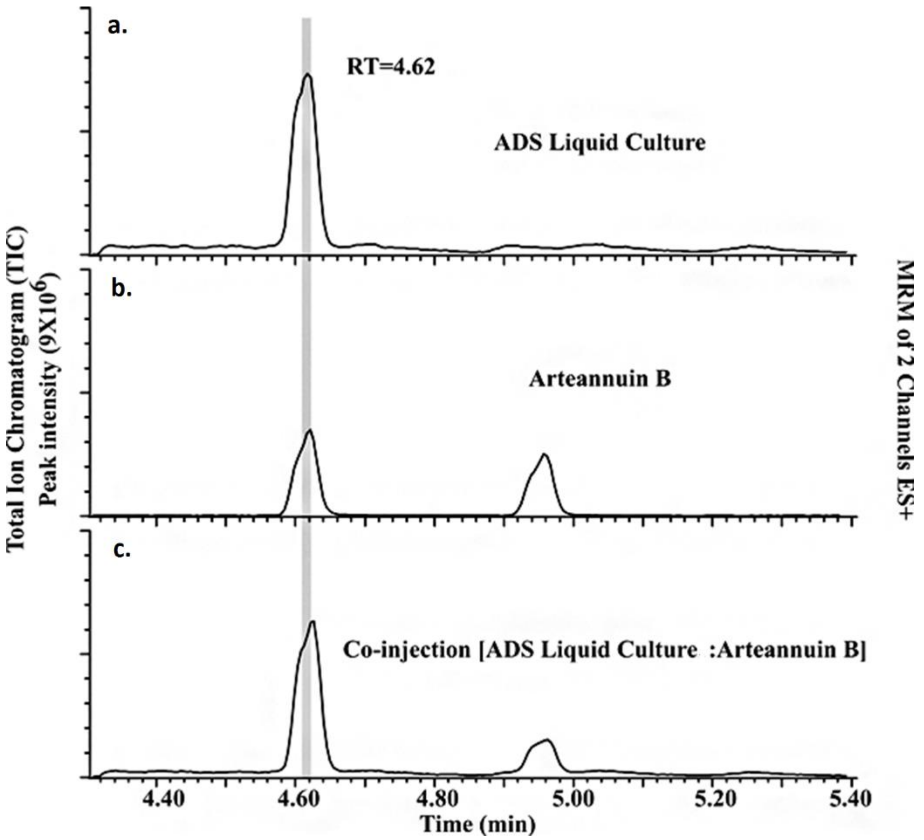

Supplemental Figure S3. UPLC-MRM-MS analysis of arteannuin B produced in (a) liquid culture of *ADS*-transgenic *P. patens*, (b) an internal standard (arteannuin B), and (c) co-injection of *ADS*-transgenic *P. patens* and internal standard (arteannuin B) as control with 4.62 retention time (RT). TIC represents the sum of MRM channels used for the detection of arteannuin B. The signal at 4.9-5 is not artemisinin, and was not identified which is also discussed in previously published work [23].

Supplemental Table S1. Primers used

	Primer Sequence	Comment
1	TCAGAATTAGATTGACATATATGTTGAAAATGGATCAAAG	Forward primer for HR 5' flanking region
2	ATTCCATTCTTGGTCAGATGAGTTTACTCTTTC	Reverse primer for HR 5' flanking region
3	TAATTCTTTCTTTTTGAGGTATATATTATCTTAGCATGG	Forward primer for HR 3' flanking region
4	ACGAAGGCCGTTCTTCCCTGG	Reverse primer for HR 3' flanking region
5	CCCTGTTGTTTGGTGTTACTTCTGCAGGTCGAAGCTAAATGGGCTAACGAAGGC	Forward primer for ADS
6	GGCGTCTCGCATATCTCATTAAAGCAGGACTCAGATGGACATCGGGTAAACCAG	Reverse primer for ADS
7	TAATGAGCATTGCATGTCTAAGTTATAAAAAATTACCAC	Forward primer for ZmUBI promoter
8	AAATAATTATAAAACATACTTGTTTATTATAATAGATAGGTACTCAAGGTTAGAGC	Reverse primer for ZmUBI promoter
9	GTCTCGCATATCTCATTAAAGCAGGAC	Forward primer for OCS terminator
10	GTTACCCGGCCGCCGTCCTCAAAAAGAAAGAATTA	Reverse primer for OCS terminator
11	CTACTCCAAAAATGTCAAAGATACAGTCTCAGAAG	Forward primer for G418 selection casette
12	ACTGGATTTTGGTTTTAGGAATTAGAAATTTTATTGATAGAAG	Reverse primer for G418 selection casette
13	GGCCCGAGGTCATTCATATGC	Forward primer for rice actin promoter
14	GTGCCATTGCTTTGAGGATAGATTTCATTCTAGAGGATCCCCGATATCTTCTACC	Reverse primer for rice actin promoter
15	ATGAAATCTATCCTCAAAGCAATGGCAC	Forward primer for CYP71ADH1-LP4/2A-ADH1
16	AGTAGCAACTTCGTCTGCTGCATTTGATCAAAACTTAATAAGGATTTTCACGCAGTCAGG	Reverse primer for CYP71ADH1-LP4/2A-ADH1
17	AGCGGCCGATCGTTCAAAC	Forward primer for NOS terminator
18	GAGACTGTATCTTTGACATTTTTGGAGTATTAGCATTCTTTCTGAAAGGGAATTCTCATG	Reverse primer for NOS terminator
19	TACTCCAAAAATGTCAAAGATACAGTCTCAGAAG	Forward primer for Hyg selection casette
20	AGTTTTGATCTTGAAAGATCTTTTATCTTTAGAGTTAAGAACTCTT	Reverse primer for Hyg selection casette
21	ACAACCAAGCGGCTTGAAACAATAG	Forward primer for AtiUBI promoter
22	GGAGAACAGAGTAGGTTTTTCGGACATCTTTTGTGTTTCGTCTTCTCTCACGTAGAAAC	Reverse primer for AtiUBI promoter
23	ATGTCCGAAAAACCTACTCTGTTCTCC	Forward primer for DBR2-LP4/2A-ALDH1
24	AGCAAGCAAGAGATGGGATTCTTGATAAGAGTCTCTTCACAGCCACGGACTGTCATAG	Reverse primer for DBR2-LP4/2A-ALDH1
25	AGAGACTCTTATCAAGAATCCCATCTCTTGC	Forward primer for Arabidopsis terminator
26	ACTGTATCTTTGACATTTTTGGAGTAGAGTTTGGTACGTCACAAACTTAAATCATTTTAC	Reverse primer for Arabidopsis terminator
27	ATCCGCCCGATCGCTAACTTC	Forward primer for qPCR-ADS
28	CTGGATCTCGTCGATCAGCTTCAAC	Reverse primer for qPCR-ADS
29	CTCTTACAGGCGAGATTGTGCTCTATC	Forward primer for qPCR-CYP71AV1
30	TCTTGCACGAGGTTCCAACACTC	Reverse primer for qPCR-CYP71AV1
31	GGAAGCATCAAAGATCGGCATTGG	Forward primer for qPCR-ADH1
32	GAGAAGTTCATCAAGCTGGATCTCCTTG	Reverse primer for qPCR-ADH1
33	CGGTTGGCTTACCTGCACG	Forward primer for qPCR-DBR2
34	AAGTCGGCATCCCCTTGTGC	Reverse primer for qPCR-DBR2
35	ATGTCAAGCGGGGCTAACGG	Forward primer for qPCR-ALDH1
36	CTTGCAGCTTTCACTGCGAGG	Reverse primer for qPCR-ALDH1

Supplemental Table S2. Optimized MRM transition settings for UPLC-MRM-MS measurement of artemisinin, artemisinic acid, dihydroartemisinic acid, artemisinic alcohol and dihydroartemisinic alcohol.

	Parent (*m/z*)	Daughter (*m/z*)	Cone voltage	Collision voltage
Artemisinin	283.19	265.22	12	14
		247.19	12	8
		219.21	12	8
Artemisinic acid	235.16	217.21	18	14
		199.25	18	16
		189.22	18	10
Dihydroartemisinic acid	237.16	163.17	16	28
		107.12	16	26
		81.10	16	18
Artemisinic alcohol	221.16	203.27	14	20
		147.09	14	10
Dihydroartemisinic alcohol	223.22	205.27	14	24
		109.13	14	14
		95.07	14	12

Supplementary Table S3. Retention time of measured metabolites by UPLC-MRM-MS, positive ionization mode.

Compound	*Retention time (minute)*
Artemisinin	4.90
Arteannuin B	4.62
Dihydroartemisinic alcohol	6.31
Artemisinic alcohol	6.17
Dihydroartemisinic acid	5.92
Artemisinic acid	6.03
